# Lidocaine and Ketamine Infusions as Adjunctive Pain Management Therapy: A Retrospective Analysis of Clinical Outcomes in Hospitalized Patients Admitted for Pain Related to Sickle Cell Disease

**DOI:** 10.3389/fpain.2022.878985

**Published:** 2022-08-04

**Authors:** Nicolas A. Zavala, Randall W. Knoebel, Magdalena Anitescu

**Affiliations:** ^1^Department of Anesthesiology, Northwestern Memorial Hospital, Chicago, IL, United States; ^2^Department of Pharmacy, The University of Chicago Medicine, Chicago, IL, United States; ^3^Department of Medicine, The University of Chicago Medicine, Chicago, IL, United States; ^4^Department of Anesthesia and Critical Care, The University of Chicago Medicine, Chicago, IL, United States

**Keywords:** opioid tolerance, lidocaine infusion, sickle cell disease, opioid dose reduction, vaso-occlusive crisis

## Abstract

**Objective:**

In this study, we aim to evaluate the efficacy of adjunctive lidocaine and ketamine infusions for opioid reduction in the treatment of sickle cell disease in patients with vaso-occlusive crisis (VOC).

**Design:**

We retrospectively reviewed a cohort of 330 adult sickle-cell crisis hospital encounters with 68 patients admitted to our institution from July 2017 to August 2018.

**Methods:**

Upon institutional IRB approval, we obtained initial data from billing records and performed chart reviews to obtain pain scores and confirm total opioid consumption. If provided by the acute pain consultation service, the patients received either a lidocaine or a ketamine infusion of 0.5–2 mg/min or 2–3 mcg/kg, respectively, for a maximum of 24–48 h. We compared the change in opioid consumption before and after infusion therapy to patients that did not receive ketamine or lidocaine.

**Results:**

Compared to patients that did not receive infusion therapy, ketamine and lidocaine accounted for respective relative decreases of 28 and 23% in average daily morphine consumption (*p* = 0.02). Patients that received either infusion were 3 to 4 times more likely to decrease their opioid consumption independent of treatment length or baseline opioid doses (*p* < 0.01). Ketamine and lidocaine therapies were not associated with change in pain scores. When a patient had multiple admissions, opioid reduction was strongly correlated with initiation of infusions in the later visits.

**Conclusion:**

Both ketamine and lidocaine infusion therapies are effective in reducing opioid consumption for patients with vaso-occlusive crisis. Lidocaine infusion is emerging as an agent for stabilizing opioid doses in VOC for patients with high daily MME.

## Introduction

Sickle cell disease (SCD) is currently estimated to affect 100,000 Americans and occurs in 1 out of every 365 African American births. Due to advances in comprehensive medical care, mortality has decreased in all age groups. From 1980 to 2009, the survival of patients 20 years of age and with sickle cell disease to rose from 50 to 85%. Subsequently, SCD prevalence among older patients is expected to rise along with comorbid conditions, including opioid tolerance and treatment-resistant pain syndromes. Moreover, Quinn et al. demonstrated that the current care of pediatric sickle cell disease allows for survival beyond 90%; as such, more than 93.5% of pediatric patients with sickle cell anemia and more than 98% of patients suffering from milder forms reach adulthood (1, 2).

In SCD, red blood cell deformation in small vessels causes pain by partial obstruction of small vessels and capillaries; the pain is often chronic and debilitating. With complete obstruction of the vessels, pain is acutely escalated and is known as vaso-occlusive crisis (VOC). The severe pain is accompanied by a significant inflammatory response that often requires hospitalization and treatment of organ failure and acute complications and such as acute chest syndrome, leg ulcers, and avascular necrosis of long bones (3).

Opioids are the mainstay of treatment for both chronic pain and acute escalations of vaso-occlusive crisis (VOC) (4). Among patients with SCD, 29% report daily pain, and most of them report pain on more than 50% of days (5). In a cohort analysis of more than 200 patients, the Pain in Sickle Cell Epidemiology Study (PiSCES) project reports that opioids are used in 75% or more of home pain days, as the 219 enrolled patients reported using opioids in 12,311 (78%) of the 15,778 home pain days. Additionally, it reports that even while adjusting for pain levels and psychosocial factors, the frequent use of opioids is correlated with higher levels of somatic symptom disorder, negative coping, and worsening mental and physical quality of life (6). By adulthood, most of the patients have experienced more than 15 years of intermittent opioid exposure (7).

In many cases, the chronic pain and undertreated acute pain in VOC may induce maladaptive changes in the central and peripheral nervous systems (8). Central sensitization predisposes patients to augmented pain signal processing by upregulation of glutamatergic NMDA-receptors in the spinal cord. Because NMDA receptor agonism is pronociceptive, this phenomenon results in hyperexcitability, hyperalgesia, and, subsequently, refractory chronic pain (9). Attempts to include multimodal analgesic techniques have been made in many inpatient protocols in an attempt to stabilize opioid consumption, decrease pain, and improve functioning (10). Our institution's suggested VOC guidelines used for this study are summarized in Table 1. However, because of the complexity of the case, treatment is often individualized for each patient, and as such, a protocol may follow all or parts of the decision tree.

**Table 1:** University of Chicago sickle cell disease vaso-occlusive inpatient pain management algorithm.

**1. Admission**	**2. 24 h from admission**		**3. Pain still unrelieved after 24 h from previous intervention**
- Continue prior to admission (PTA) lonag acting and short acting opioid - Continue PTA neuropathic analgesics - Provide non-pharmacologic pain management education - Acetaminophen 650 mg q 6 h - Ibuprofen 600 mg q 6 h; exclusion: acute kidney injury, cardiac disease - Gabapentin 300 mg TID (standing) - Hydromorphone PCA x 24 h	- Discontinue PCA - Consider co-analgesics methadonex 48 h for patient on >50 mg orla morphine equivalents - Continue acetaminophen, ibuprofen, gabapentin		- Consult acute pain service for consideration of ketamine, lidocaine or alternative techniques
	**Pain well controlled** - Start oxycodone or hydromorphone: 50% of prior 24 h consumption divided into q 4 h administration	**Pain NOT well controlled** - Start oxycodone or hydromorphone 75–100% of prior 24 h consumption divided into q 4 h administration - Start methadone (see table)	
Methadone-starting dose*		Oral morphine equivalents (24 h total)	
2.5 mg TID po		<60 mg/day	
5 mg TID po		>60 mg/day	
Consider expert consultation		>300 mg/day	

* *Concurrent opioid dose should not be escalated while receiving methadone as a co-analgesic*.

*Patients started at 7.5 mg/day can be titrated to 15 mg/day after 24 h of dosing.

Both ketamine and lidocaine are used routinely as part of multimodal perioperative analgesic protocols and have emerged as reasonable choices for attempting to relieve such poorly controlled pain. Ketamine is a potent NMDA-receptor antagonist that attenuates the central nervous system's processing of nociceptive afference. Subanesthetic doses of ketamine are analgesic for opioid-resistant or refractory cancer pain syndromes and are increasingly used in the treatment of chronic neuropathic and non-cancer pain (11–13). The role of ketamine at first presentation in the emergency department has yielded mixed results from indicating no effect on pain scores to a significant improvement 15 min after arrival when compared to patients who received morphine. In those studies, ketamine treatments were administered either as sole administration for over 15 min or as 2 doses administered for over 5 min, 20 min apart (14, 15). Studies on ketamine for dose stabilization or adjunctive therapy in refractory hospitalized patients is limited to case studies or pediatric patients (16).

Systemic effects of IV lidocaine, while less understood than its local anesthetic properties, have been demonstrated in patients with chronic neuropathic pain. Its systemic effects are believed to target dysfunctional nerves and prevent depolarization of neurons in the sodium channel. Also, IV lidocaine may prevent hypersensitization of sodium channels and block their spontaneous firing in a damaged tissue (17). In acute pain, IV lidocaine has been shown to be analgesic and anti-inflammatory and to reduce NMDA-mediated depolarization of target neurons (18). The success of systemic lidocaine has been demonstrated in hospice, perioperative, and complex regional pain syndrome patients. These patients often experience faster pain relief, have less bowel dysfunction, and require fewer opioids (19–21). While some groups in small cohorts have shown that lidocaine infusions can be helpful for some patients with VOC, they lack an “unexposed” group (22).

Our retrospective analysis uniquely compares hospitalized patients with SCD patients who were administered systemic lidocaine or ketamine infusions to those who did not. The primary goal of our study was to identify a clinically meaningful outcome in the use of either of the infusions to justify their continued study and use on patients with advanced pain syndromes. Therefore, our main outcome was decrease in average daily morphine dose when using either of the infusion therapies.

## Methods

Upon obtaining institutional IRB approval, we conducted a retrospective cohort analysis of adults with sickle cell disease admitted for vaso-occlusive crisis at the University of Chicago from January 2017 to August 2018. All opioid therapy and dose escalations were guided by the primary admitting team. When the internal medicine team treatment plan was not effective in reducing patient pain, an acute pain service consultation was initiated roughly within the first week of hospitalization, generally around day 4; however, late pain consultations (days 5–10) were also recorded and the reason for delayed consultation was unclear. If deemed appropriate by the consulting acute pain service, lidocaine or ketamine infusions were respectively administered at 0.5–2 mg/min and 2–3 mcg/kg/min until pain relief for a maximum of 48 h and a minimum of 24 h as per University of Chicago inpatient lidocaine and ketamine infusion protocols (Tables 2, 3). Infusions were stopped at 24-h period only if pain scores by NRS improved by more than 50%. The choice for the type of infusion to be administered and the time frame of its administration considered any patient-specific factors that might favor one type of infusion over the other and were in concordance with the preference of the physician treating the pain. Chart reviews were performed to obtain daily pain scores, confirm diagnoses, and verify oral morphine milligram equivalents (MMEs). In the process of retrospective analysis, data sources such as billing records provided information regarding medication prescribing and utilization, patients demographics and general hospital stay; all these data were reviewed.

**Table 2:** University of Chicago inpatient ketamine infusion protocol.

**Restrictions**	**Dosing, dispensing, and administration**	**Monitoring**
Acute pain service consultation required to initiate therapy A member of the acute pain service will order low-dose ketamine through the order set prior to initiation	Dose 0.06–0.3 mg/kg/h based on ideal body weight Starting doses: Adults: normal renal or hepatic function start at 0.12 mg/kg/h; consider starting at 0.06 mg/kg/h for BMI <18 Pediatrics: 0.06 mg/kg/h based on ideal body weight Contraindications: liver failure Change infuse rate only by acute pain service Current opioids reduced if possible during ketamine dose titration	Continuous pulse oximetry Routine vital signs, pain and sedation scores every 2 h x 1 then every 4 h for the duration of infusion Monitor closely for: blood pressure changes, mental status changes, respiratory rate <10 breaths/min, patient slow to arouse; if any of the signs occur, stop infusion and infuse acute pain service.

**Table 3:** University of Chicago inpatient lidocaine infusion protocol.

**Protocol**	**Definition**	**Restrictions**	**Dosing**	**Monitoring**
Low dose	Continuous infusion for optimal analgesia (maximum 24 h)	Ordered by acute pain service available on regular hospital units with continuous telemetry	Bolus: 1–1.5 mg/kg over 10 min, followed by a flat dose rate based on ideal body weight category <70 kg = 0.5 mg/m 70–100 kg = 0.75 mg/m 100 kg+ = 1 mg/m	Drug level monitoring First level when patient arrives in PACU, subsequent levels daily with morning labs Therapeutic blood level: 1.5–6 μg/mL Monitor for side effects every 4 h Continuous telemetry to monitor for dysrhythmia
Moderate dose	Continuous infusion > 24 h Duration of therapy > 24 h	Ordered by acute pain service available in intensive care units, PACU, and emergency department	Bolus: 1–1.5 mg/kg over 10 min, continuous after that at 0.5–3 mg/kg/h (all ideal body weight)	

Patients were excluded from analysis if there was evidence of ICU admission, invasive procedure, or cardiac disease that was determined by mention of arrhythmias, cardiomyopathy, ischemia, or heart failure in patient progress notes or problem lists. This was done because they were either contraindications to the interventions of interest or would separately require the use of opioids for sedation or post-procedural pain. We additionally omitted patients who received lidocaine and ketamine infusions on the first day of their admission, as there would be no way to establish their inpatient opioid requirements prior to the infusion.

Overall, we reviewed three separate groups: those who received ketamine infusion (KET), those who received lidocaine infusion (LID), and those who received neither infusion (SOC-standard of care). All the patients received multi-modal analgesics including opioid therapy. The SOC (standard of care) group included patients who never received either infusion during hospital stay. The KET (ketamine-infused) and LID (lidocaine-infused) groups were not combined at any point during the analysis. We decided to conduct the analysis not only on independent patients but also on hospital encounters, as the patients studied had multiple admissions to the hospital due to VOC and were treated with different multimodal analgesic regimens by multiple admission teams. Given the fact that the analgesic regimen could differ between admissions, even for the same patient, we were able to analyze each encounter as a different event, thus likely limiting both bias and possible placebo effect.

**Figure 1:**
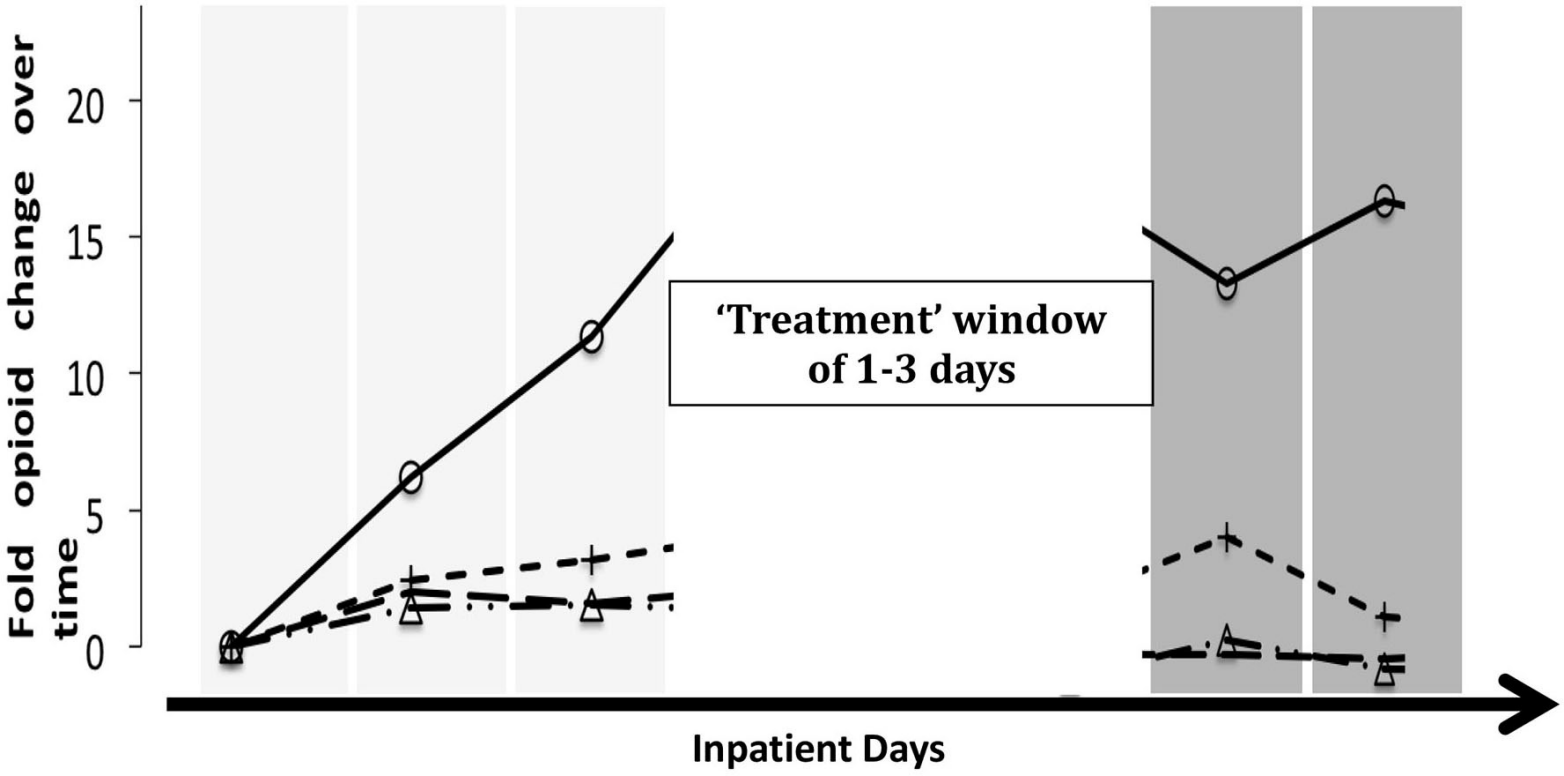
Schematic of analysis. Patient comparisons were complicated as infused patients had a window of treatment and, thus, point of reference that was not naturally occurring in non-infused patients. To account for this, we randomly chose a reference day from control encounters and randomly assigned a window (length) of treatment. In order to give a visual explanation of the way the analysis of the “no infusion” group defined the treatment window, four randomly selected patients with different trajectories for MME were chosen; as such, each line represents a patient.

As primary outcome, we analyzed the reduction of opioid use before and after the treatment with either infusion or as standard of care. For each hospital encounter, we determined opioid use before and after a “therapeutic window”. For encounters with infusion therapy, the therapeutic window coincided with the treatment, the infusion. At baseline, the average daily morphine miliequivalent (MME) dose was obtained for up to 3 days prior to the start of infusion therapy and for up to 3 days following the therapy (Figure 1). For encounters with no infusion therapy, therapeutic window start date and duration were randomly assigned, conducted in a manner that reflected the distributions of the data from the patients treated with infusion therapies. This allowed for us to make more accurate comparisons between encounters while accounting for duration of treatment as a potential major confounder. Additionally, treatment window lengths were assigned as 1, 2, or 3 hospital days. This reflects the full range of possible treatment durations.

As secondary outcomes, we aimed to identify and compare length of stay and changes in subjective pain scores before and after the treatment window in the KET (ketamine-infused), LID (lidocaine-infused), and SOC (non-infused) groups.

### Statistical Analysis

Two separate *t*-tests were conducted to compare the mean daily MME change before and after the treatment between each infused group and the same standard-care group. Initial analysis was performed using patient encounters. To compare the likelihood of dose reduction in MME after therapy, generalized estimating equations (GEEs), with opioid dose decrease (yes vs. no) as the dependent variable, were used for the analysis of direct comparisons of the effectiveness of ketamine over no infusion or lidocaine over no infusion. The GEE technique with an exchangeable correlation structure took into account the within-subject correlation structure and provided robust standard errors. GEE analyses with the same dependent variable were also conducted to identify predictors of outcome. The following variables were analyzed: age, sex, treatment window length, baseline opioid dose, day of stay, and weather any NSAIDs, membrane stabilizers, methadone, or acetaminophen had been administered in the days leading to the treatment window. Because of the difficulty of converting methadone to morphine equivalents due to its unpredictable half-life, we decided to account for its use and dosing separately from the opioid use.

We lastly performed a separate analysis to estimate the adjusted risk ratio by stratified analysis and using a log-binomial model with a 2:1 matched group (conditional logistic regression) based on age, sex, and baseline MME. Adjusted relative risk was also calculated because of the tendency of odds ratios to overestimate the impact of predictors on common events (23). All the analyses were conducted on anonymized data with Stata.

## Results

Overall, 330 hospitalizations of 68 patients met the study criteria. Of these encounters, ketamine and lidocaine infusions were administered in 28 and 29 of the admissions for 16 and 21 unique patients, respectively. Twenty-seven patient encounters were omitted as a result of the exclusion criteria. During four separate encounters, patients received both lidocaine and ketamine (not concomitantly). They were not included in the analysis, as the reason for switching between infusions was not clear (adverse event vs. futility). Both infusion groups, KET (ketamine infusions) and LID (lidocaine infusions), were different from the SOC (standard of care) group, as they had a greater proportion of females who were younger and consumed more morphine equivalents per day at baseline. Additionally, when compared to patients receiving lidocaine, patients receiving ketamine infusions typically had longer hospital stays and received ketamine 1.5 days later than patients who received lidocaine (Table 4).

**Table 4:** Baseline characteristics of patient encounters.

	**Neither**	**Ketamine**	**Lidocaine**
Number of encounters	273	28	29
Female (83)	0.63	0.87*	0.71
Median age	30 (23–37)	27 (23–29)*	26 (21–30)*
Median daily MME	240 (140–415)	820 (225–2,100)*	492 (251–860)*
Median day of administration	N/A	5.5 (3–8.5)*	4 (3–8)
Median length of stay	9 (6–12)	12.5 (9–17.5)*	9 (7–16)

*A total of 330 encounters from 68 patients were identified from January 2017 to July 2018. Encounters with ketamine and lidocaine infusions represent 16 and 21 patients, respectively*.

*p < 0.05 in the identified baseline characteristic when compared to the control group.

A total of 299 patient encounters from 68 unique patients remained for analysis. Twenty-four encounters received ketamine (16 unique patients, eight encounters were repeat admissions), 24 encounters received lidocaine (21 unique patients, three encounters were repeat admissions). A total of 251 inpatient encounters had no administration of either infusion drug (42 unique patients).

**Figure 2:**
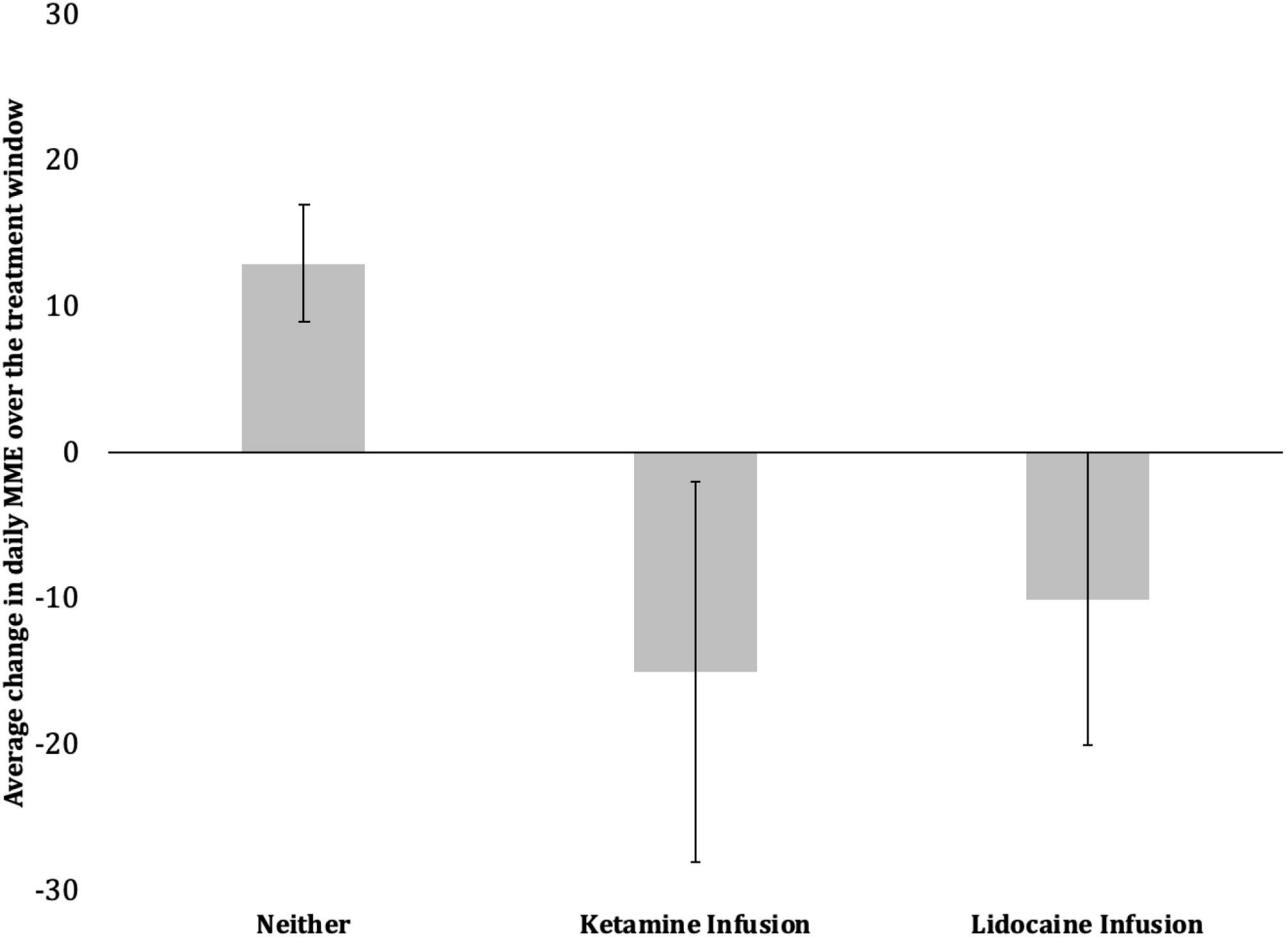
Comparison of change in average daily oral morphine equivalents between encounters with infusion therapy and those receiving standard care. Data are mean change in opioid consumption over treatment window (± 1 SE). The ketamine group and the lidocaine group are significantly different from the standard-care group (p = 0.02, p = 0.03; t-test).

Doses of morphine daily equivalents were calculated taking in consideration all opioids administered to patients with a wide range of daily opioid use at baseline. After the treatment window, the mean change in MME for the SOC (standard-care), KET (ketamine infusion group), and LID (lidocaine infusion group) increased by +13% (± 4) in the SOC group and decreased by −15% (± 13), and −10% (± 10) in KET and LID, respectively. It was statistically significant (*p* < 0.05) (Figure 2).

The KET (ketamine infusion group) patients who received ketamine infusions during their admissions were 65% less likely to increase their daily MME after the treatment window (ARR 0.35; 95% CI [0.12–0.83], *p* < 0.01) when compared with SOC (standard of care group) patients who never received either of the infusions. Similarly, the LID (lidocaine infusingroup) patients who received lidocaine infusions were 73% less likely to increase their opioid consumption (ARR 0.27; 95% CI [0.21–0.72], *p* < 0.01) when compared with the SOC (standard of care group) patients who never received either of the infusions (Table 5).

**Table 5:** Results of intergroup and conditional logistic regression analysis.

	**Neither**	**Ketamine**	**Lidocaine**	***p***
	**(SOC)**	**Infusion (KET)**	**Infusion (LID)**	
Encounters (n)	251	24	24	
Mean MME change (SE)	13% (± 4)	−15% (± 13)	−10% (± 10)	0.02
**Decrease in MME after treatment window**				
Opioid decrease frequency	0.52	0.67	0.67	0.1
Unadjusted RR	-	1.45	1.45	-
AOR [95%CI]	-	4.76 [1.4–17]	6.7 [1.8–25]	<0.01
ARR [95% CI]	-	2.9 [1.2–8.3]	3.7 [1.4–4.8]	<0.01

*Data are based on the relative average change (decrease) in daily MME over the treatment window. Data are adjusted for age, sex, days of patient stay, mean opioid consumption, and co-administration of acetaminophen, nonsteroidal anti-inflammatory drugs (NSAIDs), membrane stabilizers, or methadone.AOR, adjusted odds ratio; ARR, adjusted relative risk; SE, standard error*.

On average, patients that had an increase in MME (*n* = 141) after the treatment window had 5.83 (± 0.73) days until hospital discharge, whereas those who had a decrease in MME (*n* = 158) had, on average, 4.54 (±0.73) days until discharge. The difference among the groups was significant (*p* < 0.01). However, the KET and LID groups were not independently associated a shortened stay or change in pain score during the therapeutic window.

When evaluating individual patients using the GEE population-averaged model, we calculated the correlation of the treatment groups, KET and LID, with subsequent hospital visits. By analyzing 211 individual complete observations summarizing 59 patients that had two or more admissions, the results showed that patients with multiple admissions had later hospital visits with significantly higher probability of decreasing opioid consumption for both KET (ketamine infusion) and LID (lidocaine infusion) groups compared to SOC (standard of care) group respectively (Table 6), perhaps due to introducing infusions therapies earlier in the pain treatment allgorhythm.

**Table 6:** Generalized estimation equation (GEE) model with exchangeable correlation structure.

**Opoid**	**Odds**	**Standard**	**z**	**P > z**	**95% confidence interval**	
**decrease**	**ratio**	**error**				
Ketamine	1.45	0.22	2.39	0.02	1.07	1.98
Lidocaine	1.24	0.12	2.18	0.03	1.02	1.51

*Results of interaction of treatment between primary outcome, decrease in opioid consumption, number of patient hospital admissions, and administration of either infusion (ketamine or lidocaine) when compared to those that received neither*.

## Discussion

With the advent of routine pneumococcal vaccination and penicillin prophylaxis in the pediatric population, most patients with SCD have begun to live and thrive well past age 20 (3). Moreover, transcranial doppler (TCD), as a screening test for stroke in children with SCD, is emerging as a major factor leading to improvement in life expectancy during childhood. Upon reaching adulthood, painful vaso-occlusive crises become the number one reason for hospital admission. It is believed that VOC episodes can precipitate further complications such as ulcers, neuropathy, cholestasis, and organ failure (24). Thus, abortion of these episodes is considered paramount. In addition to nonsteroidal anti-inflammatory agents, opioid analgesics are the key component of pain management in hospitalized patients (25).

Both earlier and higher opioid doses in the emergency department were shown to be associated with shorter hospital stays (26). Still, the pain of many patients is refractory to standard abortive therapy. Ketamine infusion and nitric oxide have been demonstrated to provide some immediate pain relief in the emergency department (15, 25–27). While there has been no beneficial effect shown for nitric oxide on refractory pain, many case reports exist on the successful use of ketamine especially with longer infusion treatments rather than single administration (16, 28–30).

In the search for multimodal abortive agents, infusion therapies, traditionally used in perioperative periods, may also prove beneficial for patients with SCD admitted for VOC.

Our study is unique in that we specifically compare, albeit retrospective, the use of ketamine and lidocaine infusions with encounters that received neither. While we suggest ketamine infusions to be an emerging modality for the treatment of sickle cell pain, thus far, there is little evidence to support the use of systemic lidocaine in this setting on general adult population (31). Reports on the efficacy of both lidocaine and ketamine infusions in adolescents with vaso-occlusive crisis have emerged recently (32). Similarly, lidocaine infusions have been shown to reduce opioid consumption in pediatric hematology and oncology patients with refractory pain (33).

From our results, there is evidence that both ketamine and lidocaine are equally efficacious in stabilizing, and even reducing, the daily demand for opioid management independent of baseline opioid doses, length of therapy, and co-administration of other analgesics, especially in later visits of multiple admissions. While reduction in daily MME was associated with shorter duration until discharge, this effect was not mediated by ketamine or lidocaine infusion. Similarly, neither infusion had any effect on change in daily numerical pain scores. However, reducing opioid use may have been essential in further mitigating dose escalation and development of tolerance and opioid-induced hyperalgesia, thus allowing patients to progress, with proper support and medical treatment, toward overcoming the vaso-occlusive crisis and to subsequent hospital discharge.

Of note, our analysis showed that both the LID and KET groups appear to have had longer hospital stays than the SOC group; as the infusion groups do seem to represent a small percentage of all analyzed hospitalizations for patients with VOC, the KET and LID groups may indeed indicate a group of patients with more refractory pain that may benefit from initiation of infusion therapies earlier in their hospitalization course.

Our study has limitations. Its greatest limitations are those inherent to its single-center retrospective nature and lack of prospective controls. Importantly, while patients are hospitalized and treatment was at the primary care providers' discretion, we can neither determine exactly why certain individuals who qualified for ketamine or lidocaine infusions did not receive them, nor can we determine exactly why doses of opioids were manipulated. In addition, from reviewing of medical records, it did not seem that the choosing of the lidocaine or ketamine as a treatment was correlated with the state of “responder” to either of these medications but it was left at the discretion of the acute pain service attending and based on drug availability. The validity of our analysis of daily morphine requirements hinges on the assumption that subjective pain is treated with nearly on-demand dosing of opioids. In other words, as pain subsides, so should doses. Our study does not take into account social and psychological aspects related to long-term opioid use.

Another limitation of our retrospective review is the small number of subjects. While there were many encounters, only 68 individual patients were actually identified and 59 were included in the final GEE analysis. The correlation between infusion treatment and number of visits seems to show that patients with multiple admissions seem to rely more on the infusions to decrease opioid consumption during later hospitalization and not the initial ones. In these instances, our findings suggest that either infusion, ketamine or lidocaine, would be equally effective in maintaining and/or decreasing high opioid doses employed in hard to treat crises.

Multiple providers with various treatment plans made our study challenging to interpret as well. With involvement of the consulting pain service and initiation of infusions, data recorded in electronic medical record during those treatments may have been more easily retrievable than in the “no infusion”, SOC or standard of care groups, as acute pain service note has standardized elements to identify pain management treatment outcomes. This was another challenge of our study.

As there is a strong correlation between decrease in opioid consumption and initiation of either infusion the more frequent a patient is admitted to the hospital, we hypothesize that our treatments do have a stabilizing effect on opioid dose, especially in patients with significant exposure during earlier visits. It would be therefore worth investigating the initiation of lidocaine or ketamine infusions early in the admission process to perhaps limit the further use of opioids in this challenging patient population. Therefore, our institution initiated a multidisciplinary task force to develop a comprehensive clinical pathway for the treatment of VOC (Table 7). As such, this protocol suggests early acute pain service interventions as the sole service responsible for initiation of infusion therapy. Thus, based on this pathway, lidocaine and ketamine infusions can be initiated as early as day 2 of hospital stay, as their use would be dictated by the patients' response to conventional and standard of care treatments; per protocol, if assessment during the first day of hospitalization at three3 time points, 8 h. apart, does not render a patient significant pain relief, acute pain consultation and subsequent initiation of infusion therapies would be indicated; this will allow starting of the rescue infusion as early as the 2nd day (in some cases 3rd day) of hospitalization, allowing for, hopefully, better pain control in patients with painful VOC.

**Table 7:**
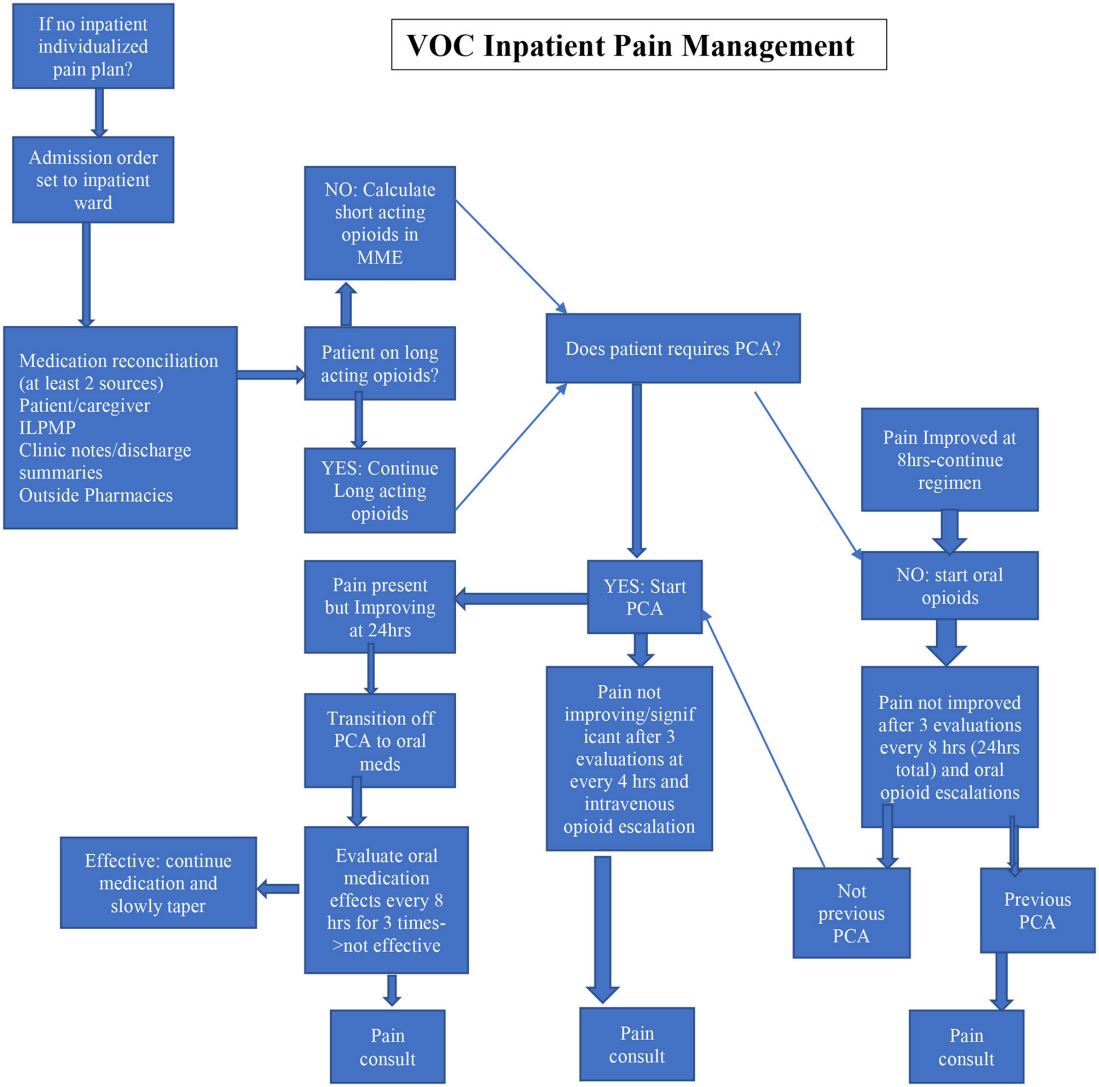
Vaso-occlusive (VOC) crisis inpatient pain management.

*Decision tree shows quick escalation to inpatient pain consult if opioid regimen is ineffective*.

Despite its limitations, because prospective studies on ketamine are difficult to conduct as patients can easily identify the treatment received due to side effects encountered, our retrospective review provided evidence by simple and robust analysis for the continued use and study on lidocaine and ketamine infusions in VOCs. Our analysis seems to suggest that both ketamine and lidocaine have similar effects in decreasing opioids when initiated in refractory and painful VOC. More research is needed to identify proper doses and outcomes in patients with SCD admitted for painful occlusive crises.

## Conclusions

Patients with SCD often reach adulthood as opioid-tolerant adults due to their long-term use of opioids to treat chronic pain and acute exacerbations. When opioid dose escalation alone does not provide relief during painful crises, multimodal analgesia is required; therefore, every hospital that treats SCD should have such protocols for these events. While ketamine has been used before in VOC, lidocaine has been studied more on pediatric and adolescent patients with VOC (32–34). This retrospective study identifies intravenous lidocaine as a potential analgesic similar to IV ketamine to treat acute opioid dose escalations in painful unrelenting VOC; both agents seem to have the same analgesic and stabilizing effects on opioid doses. Moreover, our study shows that lidocaine and ketamine infusions have higher probability of reducing opioid use in subsequent later admission visits. With its significant limitations as a retrospective and small study, our analysis still remains unique as it does compare the 2 infusion treatments with the no-infusion group. Moreover, our findings raise the question of initiating the infusions early in the admission and treatment process to possibly limit subsequent opioid exposure. Although we showed good results in opioid reduction using infusion therapies with either medication, future studies may be needed to prospectively explore the role of lidocaine and ketamine infusions in the treatment of refractory crisis or even as a maintenance treatment in SCD.

## Data Availability Statement

The raw data supporting the conclusions of this article will be made available by the authors, without undue reservation.

## Ethics Statement

The studies involving human participants were reviewed and approved by University of Chicago. Written informed consent for participation was not required for this study in accordance with the national legislation and the institutional requirements.

## Author Contributions

NZ conducted the research and wrote the manuscript. RK contributed to manuscript preparation and conducted the research. MA conducted the research, led the team, and wrote the manuscript. All authors contributed to the article and approved the submitted version.

## Conflict of Interest

The authors declare that the research was conducted in the absence of any commercial or financial relationships that could be construed as a potential conflict of interest.

## Publisher's Note

All claims expressed in this article are solely those of the authors and do not necessarily represent those of their affiliated organizations, or those of the publisher, the editors and the reviewers. Any product that may be evaluated in this article, or claim that may be made by its manufacturer, is not guaranteed or endorsed by the publisher.

## References

[1] Quinn CT Rogers ZR McCavit TL Buchanan GR. Improved survival of children and adolescents with sickle cell disease. Blood. (2010) 115:3447–52. 10.1182/blood-2009-07-233700 20194891 PMC2867259

[2] Dampier C Palermo TM Darbari D Hassell K Smith W Zempsky W. AAPT diagnostic criteria for chronic sickle cell disease pain. J Pain. (2017) 18:490–8. 10.1016/j.jpain.2016.12.016 28065813

[3] Ballas SK Gupta K Adams-Graves P. Sickle cell pain: a critical reappraisal. Blood. (2012) 120:3647–56. 10.1182/blood-2012-04-383430 22923496

[4] Yawn BP Buchanan GR Afenyi-Annan AN Ballas SK Hassell KL James AH. Management of sickle cell disease: summary of the 2014 evidence-based report by expert panel members. JAMA. (2014) 312:1033–48. 10.1001/jama.2014.10517 25203083

[5] Smith WR Penberthy LT Bovbjerg VE McClish DK Roberts JD Dahman B. Daily assessment of pain in adults with sickle cell disease. Ann Intern Med. (2008) 148:94–101. 10.7326/0003-4819-148-2-200801150-00004 18195334

[6] Smith WR McClish DK Dahman BA Levenson JL Aisiku IP de ACV. Daily home opioid use in adults with sickle cell disease: the PiSCES project. J Opioid Manag. (2015) 11:243–53. 10.5055/jom.2015.0273 25985809

[7] Aisiku IP Smith WR McClish DK Levenson JL Penberthy LT Roseff SD. Comparisons of high versus low emergency department utilizers in sickle cell disease. Ann Emerg Med. (2009) 53:587–93. 10.1016/j.annemergmed.2008.07.050 18926599

[8] den Boer C Dries L Terluin B van der Wouden JC Blankenstein AH van Wilgen CP. Central sensitization in chronic pain and medically unexplained symptom research: a systematic review of definitions, operationalizations and measurement instruments. J Psychosom Res. (2019) 117:32–40. 10.1016/j.jpsychores.2018.12.010 30665594

[9] Gungor S Fields K Aiyer R Della Valle AG Su EP. Incidence and risk factors for development of persistent postsurgical pain following total knee arthroplasty: a retrospective cohort study. Medicine. (2019) 98. 10.1097/MD.0000000000016450 31305475 PMC6641667

[10] Klyne DM Moseley GL Sterling M Barbe MF Hodges PW. Are signs of central sensitization in acute low back pain a precursor to poor outcome?. J Pain. (2019) 20:994–1009. 10.1016/j.jpain.2019.03.001 30853506

[11] Jonkman K van de Donk T Dahan A. Ketamine for cancer pain: what is the evidence? Curr Opin Support Palliat Care. (2017) 11:88–92. 10.1097/SPC.0000000000000262 28306568

[12] Orhurhu V Orhurhu MS Bhatia A Cohen SP. Ketamine infusions for chronic pain: a systematic review and meta-analysis of randomized controlled trials. Anesth Analg. (2019) 129:241–54. 10.1213/ANE.0000000000004185 31082965

[13] Maher DP Chen L Mao J. Intravenous ketamine infusions for neuropathic pain management: a promising therapy in need of optimization. Anesth Analg. (2017) 124:661–74. 10.1213/ANE.0000000000001787 28067704

[14] Sin B Tatunchak T Paryavi M Olivo M Mian U Ruiz J. The use of ketamine for acute treatment of pain: a randomized, double-blind, placebo-controlled trial. J Emerg Med. (2017) 52:601–8. 10.1016/j.jemermed.2016.12.039 28279542

[15] Miller JP Schauer SG Ganem VJ Bebarta VS. Low-dose ketamine vs morphine for acute pain in the ED: a randomized controlled trial. Am J Emerg Med. (2015) 33:402–8. 10.1016/j.ajem.2014.12.058 25624076

[16] Zempsky WT Loiselle KA Corsi JM Hagstrom JN. Use of low-dose ketamine infusion for pediatric patients with sickle cell disease-related pain: a case series. Clin J Pain. (2010) 26:163–7. 10.1097/AJP.0b013e3181b511ab 20090444

[17] de Oliveira CM Issy AM Sakata RK. Intraoperative intravenous lidocaine. Rev Bras Anestesiol. (2010) 60:325–33. 10.1016/S0034-7094(10)70041-6 20682165

[18] Masic D Liang E Long C Sterk EJ Barbas B Rech MA. Intravenous lidocaine for acute pain: a systematic review. Pharmacotherapy. (2018) 38:1250–9. 10.1002/phar.2189 30303542

[19] Groudine SB Fisher HA Kaufman RP Patel MK Wilkins LJ Mehta SA. Intravenous lidocaine speeds the return of bowel function, decreases postoperative pain, and shortens hospital stay in patients undergoing radical retropubic prostatectomy. Anesth Analg. (1998) 86:235–9. 10.1213/00000539-199802000-00003 9459225

[20] Koppert W Weigand M Neumann F Sittl R Schuettler J Schmelz M. Perioperative intravenous lidocaine has preventive effects on postoperative pain and morphine consumption after major abdominal surgery. Anesth Analg. (2004) 98:1050–5. 10.1213/01.ANE.0000104582.71710.EE 15041597

[21] Fitzpatrick BM Mullins ME. Intravenous lidocaine for the treatment of acute pain in the emergency department. Clin Exp Emerg Med. (2016) 3:105–8. 10.15441/ceem.15.103 27752626 PMC5051607

[22] Nguyen NL Kome AM Lowe DK Coyne P Hawks KG. Intravenous lidocaine as an adjuvant for pain associated with sickle cell disease. J Pain Palliat Care Pharmacother. (2015) 29:359–64. 10.3109/15360288.2015.1082009 26654408

[23] McNutt LA Wu C Xue X Hafner JP. Estimating the relative risk in cohort studies and clinical trials of common outcomes. Am J Epidemiol. (2003) 157:940–3. 10.1093/aje/kwg074 12746247

[24] Lovett PB Sule HP Lopez BL. Sickle cell disease in the emergency department. Hematol Oncol Clin North Am. (2017) 31:1061–79. 10.1016/j.hoc.2017.08.009 29078924

[25] Ballas SK Kesen MR Goldberg MF Lutty GA Dampier C Osunkwo I. Beyond the definitions of the phenotypic complications of sickle cell disease: an update on management. ScientificWorld J. (2012) 2012:949535. 10.1100/2012/949535 22924029 PMC3415156

[26] Payne J Aban I Hilliard LM Madison J Bemrich-Stolz C Howard TH. Impact of early analgesia on hospitalization outcomes for sickle cell pain crisis. Pediatr Blood Cancer. (2018) 65:e27420. 10.1002/pbc.27420 30151977 PMC6192851

[27] Head CA Swerdlow P McDade WA Joshi RM Ikuta T Cooper ML. Beneficial effects of nitric oxide breathing in adult patients with sickle cell crisis. Am J Hematol. (2010) 85:800–2. 10.1002/ajh.21832 20799359

[28] Ikuta T Thatte HS Tang JX Mukerji I Knee K Bridges KR. Nitric oxide reduces sickle hemoglobin polymerization: potential role of nitric oxide-induced charge alteration in depolymerization. Arch Biochem Biophys. (2011) 510:53–61. 10.1016/j.abb.2011.03.013 21457702 PMC3889650

[29] Gladwin MT Kato GJ Weiner D Onyekwere OC Dampier C Hsu L. Nitric oxide for inhalation in the acute treatment of sickle cell pain crisis: a randomized controlled trial. JAMA. (2011) 305:893–902. 10.1001/jama.2011.235 21364138 PMC3403835

[30] Nobrega R Sheehy KA Lippold C Rice AL Finkel JC Quezado ZMN. Patient characteristics affect the response to ketamine and opioids during the treatment of vaso-occlusive episode-related pain in sickle cell disease. Pediatr Res. (2018) 83:445–54. 10.1038/pr.2017.197 28902183

[31] Neri CM Pestieau SR Darbari DS. Low-dose ketamine as a potential adjuvant therapy for painful vaso-occlusive crisis in sickle cell disease. Paediatr Anaesth. (2013) 23:684–9. 10.1111/pan.12172 23565738

[32] Puri L Morgan KJ Anghelescu DL. Ketamine and lidocaine infusions decrease opioid consumption during vaso-occlusive crisis in adolescents with sickle cell disease. Curr Opin Support Palliat Care. (2019) 13:402–7. 10.1097/SPC.0000000000000437 31157658

[33] Anghelescu DL Morgan KJ Frett MJ Wu D Li Y Han Y. Lidocaine infusions and reduced opioid consumption-Retrospective experience in pediatric hematology and oncology patients with refractory pain. Pediatr Blood Cancer. (2021) 68:e29215. 10.1002/pbc.29215 34264551 PMC8601594

[34] Anghelescu DL Ryan S Wu D Morgan KJ Patni T Li Y. Low-dose ketamine infusions reduce opioid use in pediatric and young adult oncology patients. Pediatr Blood Cancer. (2022) 4:e29693. 10.1002/pbc.29693 35373875 PMC9329174

